# Improved targeting of the 16S rDNA nanopore sequencing method enables rapid pathogen identification in bacterial pneumonia in children

**DOI:** 10.3389/fcimb.2022.1001607

**Published:** 2023-01-09

**Authors:** Yinghu Chen, Lingfeng Mao, Dengming Lai, Weize Xu, Yuebai Zhang, Sihao Wu, Di Yang, Shaobo Zhao, Zhicong Liu, Yi Xiao, Yi Tang, Xiaofang Meng, Min Wang, Jueliang Shi, Qixing Chen, Qiang Shu

**Affiliations:** ^1^ The Children’s Hospital, Zhejiang University School of Medicine, Hangzhou, China; ^2^ National Clinical Research Center for Child Health, Hangzhou, China; ^3^ Joint Research Center for Molecular Diagnosis of Severe Infection in Children, Binjiang Institute of Zhejiang University, Hangzhou, China

**Keywords:** nanopore sequencing, 16S rDNA, bacterial pneumonia, BALF, prospective study

## Abstract

**Objectives:**

To develop a rapid and low-cost method for 16S rDNA nanopore sequencing.

**Methods:**

This was a prospective study on a 16S rDNA nanopore sequencing method. We developed this nanopore barcoding 16S sequencing method by adding barcodes to the 16S primer to reduce the reagent cost and simplify the experimental procedure. Twenty-one common pulmonary bacteria (7 reference strains, 14 clinical isolates) and 94 samples of bronchoalveolar lavage fluid from children with severe pneumonia were tested. Results indicating low-abundance pathogenic bacteria were verified with the polymerase chain reaction (PCR). Further, the results were compared with those of culture or PCR.

**Results:**

The turnaround time was shortened to 6~8 hours and the reagent cost of DNA preparation was reduced by employing a single reaction adding barcodes to the 16S primer in advance. The accuracy rate for the 21 common pulmonary pathogens with an abundance ≥ 99% was 100%. Applying the culture or PCR results as the gold standard, 71 (75.5%) of the 94 patients were positive, including 25 positive cultures (26.6%) and 52 positive quantitative PCRs (55.3%). The median abundance in the positive culture and qPCR samples were 29.9% and 6.7%, respectively. With an abundance threshold increase of 1%, 5%, 10%, 15% and 20%, the test sensitivity decreased gradually to 98.6%, 84.9%, 72.6%, 67.1% and 64.4%, respectively, and the test specificity increased gradually to 33.3%, 71.4%, 81.0%, 90.5% and 100.0%, respectively.

**Conclusions:**

The nanopore barcoding 16S sequencing method can rapidly identify the pathogens causing bacterial pneumonia in children.

## Introduction

Next-generation sequencing (NGS) was first reported for the diagnosis of neuroleptospirosis in 2014 (Wilson et al., 2014). Subsequently, the NGS has been tentatively applied in detecting pathogens causing infection, such as the coronavirus disease 2019 (COVID-19) and plague (Zhu et al., 2020; Stefan et al., 2016). However, due to the high cost and long turnaround time, NGS is usually applied to detect pathogens causing infections with treatment failure rather than early-stage severe infections in clinical setting (Consensus et al., 2020; Hutchins et al., 2019). For children with severe bacterial infection, rapid pathogen identification is important for the administration of appropriate antibiotics. Nanopore sequencing technology, with the advantages of long reads, and real-time sequencing and analysis, makes it possible to quickly detect and diagnose local pathogen (Sedlazeck et al., 2018; Shiota et al., 2018). Several studies on the application of nanopore sequencing in pathogen diagnosis have been reported (Charalampous et al., 2019; Leggett et al., 2020; Baldan et al., 2021; Zhao et al., 2021). Besides, nanopore sequencing may be used to screen for drug resistance mutations and virulence genes of pathogens (Zhou et al., 2021; Ferreira et al., 2021).

In this study, we developed a nanopore barcoding 16S rDNA sequencing (NB16S-seq) method, which was a 16S full-length gene amplification with barcoded primers prior to targeted third-generation sequencing (TGS). The amplification of full-length 16S rDNA improves the sensitivity and decreases the limit of detection. In addition, the barcode sequence is added to the primer in advance, and this optimization can detect the amplification of full-length 16S rDNA and the ligation of barcodes in one step. The above features not only reduce the reagents cost of end-repair and dA-tailing for all samples to one pooled sample but also avoid the aerosol contamination from the barcode addition step. This prospective study was carried out to ascertain the significance of the NB16S-seq in the identification of bacterial pneumonia pathogens.

## Methods

### Study design and participants

This was a prospective study on an improved 16S rDNA nanopore sequencing method. Before testing clinical samples, the 21 common pathogens of bacterial pneumonia (7 reference strains and 14 clinical isolates) were sequenced by the NB16S-seq to establish its accuracy.

Bronchoalveolar lavage fluid (BALF) (5 ml) from patients with pulmonary infection was collected in the respiratory endoscopy room between December 2021 and January 2022, and pulmonary infection was confirmed by either pulmonary X-ray or CT. The BALF was sent for culture, NB16S-seq test and *M. pneumonia* RNA detection. Pathogens with negative cultures and positive NB16S-seq (abundant ≥ 5.0%) were verified by quantitative polymerase chain reaction (qPCR). If only one pathogen was detected and its abundance was between 1% and 5%, the qPCR verification was also performed.

The gold standard of the BALF testing is either culture or qPCR. We collected the following data: age, sex, symptoms, diagnosis, antibiotics use, imaging, and duration from admission to sequencing, etc. Routine clinical microbiological investigation included the BALF culture and detection of routine respiratory virus (including respiratory syncytial virus, adenovirus, influenza A and B, and parainfluenza A, B and C) antigens from nasopharyngeal swabs with Rapid Antigen Detection Kits (Kaibili, Hangzhou Innovation Biotechnology Co., Ltd., Hangzhou, China).

### Definitions

‘Respiratory pathogens’ or ‘pathogens’ were defined in this study as common causes of respiratory infection to differentiate them from commensal organisms. The respiratory pathogens identified in this study were *S. pneumoniae, H. influenzae, M. catarrhalis, M. pneumoniae, S. aureus, S. agalactiae, L. gormanii, B. pertussis, P. aeruginosa, S. marcescens*, and *A. baumannii*.

### DNA extraction

Samples were processed in the genetic diagnostic center. The BALF was collected and stored at 4 °C before testing. The 7 reference strains and 13 clinically isolated strains were inoculated on Columbia blood plate culture medium for DNA extraction. These strains were identified by matrix-assisted laser desorption ionization time-of-flight mass spectrometry (MALDI-TOF MS; Bruker Daltoniks, Germany) before DNA extraction. The QIAamp^®^ DNA Mini Kit (QIAGEN, Germany) was used for the purification of microbial DNA. BALF (300μL) was first centrifuged at 13,000 rpm for 5 min to remove the supernatant, and then, the precipitate was suspended in normal saline (200 μL). The mixture was added to buffer ATL (180 μL) and proteinase K (20 μL), mixed by vortexing and incubated at 56 °C for 30 min at 500 rpm in a thermomixer (Eppendorf, Germany). For the 21 bacterial strains, 200 μL of sample (1×10^8^ CFU/ml), which was directly added to buffer ATL (180 μL) and proteinase K (20 μL), was incubated at 56 °C for 30 min at 500 rpm in a thermomixer after mixing well. The following experimental steps were carried out according to the manufacturer’s instructions.

### 16S rDNA full-length gene amplification and nanopore sequencing

The concentration of isolated DNA from samples was measured with a Qubit dsDNA HS Assay Kit and a Qubit 4.0 Fluorometer (Thermo Fisher, USA) according to the instructions of the manufacturer. A total of 5~10 ng of DNA template (10 μL) was used for 16S rDNA full-length sequence amplification (primers: 5’-barcode-27F-AGAGTTTGATCMTGGCTCAG-3’, 5’-barcode-1492R-CGGTTACC TTGTTACGACTT-3’) with 25 μL of Q5 Hot Start High-Fidelity 2X Master Mix (M0494L, New England Biolabs, USA) and 14.5 μL of nuclease-free water (New England Biolabs, USA). PCR amplicons were visualized by 1% agarose gel electrophoresis, followed by purification using 1x AMPure XP beads (Beckman Coulter, USA), and the DNA concentration was measured with a Qubit 4.0 Fluorometer. 1~48 amplified DNA was pooled in equimolar amounts. The pooled DNA was end-repaired and dA-tailed with NEBNext FFPE DNA Repair Mix (New England Biolabs, USA) and NEBNext Ultra II End Repair/dA-Tailing Module (New England Biolabs, USA), followed by purification with AMPure XP beads (Beckman Coulter, USA). Then, the nanopore sequencing was performed using the ligation sequencing kit SQK-LSK110 (Oxford Nanopore Technologies, UK), and the DNA library was loaded into an R9.4.1 flow cell (Oxford Nanopore Technologies, UK) for sequencing for 1~3 hours in the GridION platform according to the sample number. In every sequencing process run, negative process controls were added to the DNA extraction and amplification steps with sterile nuclease-free water to monitor contamination from the environment.

### Bioinformatics and pathogen identification

First, raw reads were demultiplexed with an in-house script to eliminate interference from error splitting. Porechop was used to trim the adapter and barcode on the end of the reads (Wick et al., 2018). Seqkit was used to filter reads, retaining those with a size range of 1.3–1.6 kb and qscore ≧ 12 (Shen et al., 2016). To identify the pathogen, clean reads were mapped to the 16S database collected from NCBI and GTDB using minimap2 v2.17 (Li H, 2018) with the parameter “-ax map-ont”, and the program samtools v1.10 (Danecek et al., 2021) was used to remove unmapped, nonunique mapped and multiple-mapped reads with the parameter “view -F2308”. The species abundance in the sample were incorporated into the biological classification according to the NCBI taxonomy tree. The pathogen identification was determined according to the matching between the species abundance in the sample and the guide for pathogens in the Manual of Clinical Microbiology, 12^th^ edition. The thresholds used in this study for pathogen identification were defined as follows: the valid read number was greater than 5000, and the reliable species abundance for pathogen identification was 1%. To reduce the impact of similar homologous segments of the same species and miscellaneous bacteria in the environment on the analytical results, we tabulated bacterial species that were frequently cultured in the BALF samples into a table (for details, see **Supplementary Table 1**). If the test bacteria met the following conditions, they were considered “detected bacteria”: ① the abundance of detected bacteria was more than 1% and the species was in the above mentioned high-frequency bacterial table; ② if not in the **Supplementary Table 1**, at most two species with abundance ≥5% were reported in the same genus; and ③ if there were more than ten species not in the **Supplementary Table 1**, they were classified according to their abundance, and only the first ten species were reported.

### Statistical analysis

Stata 15 software was used for ROC analysis, and the sensitivity, specificity and area under the ROC curve were calculated under different cross-sections. Data are expressed as the median (interquartile range).

## Results

### Rapid NB16S-seq test method

**Figure 1:**
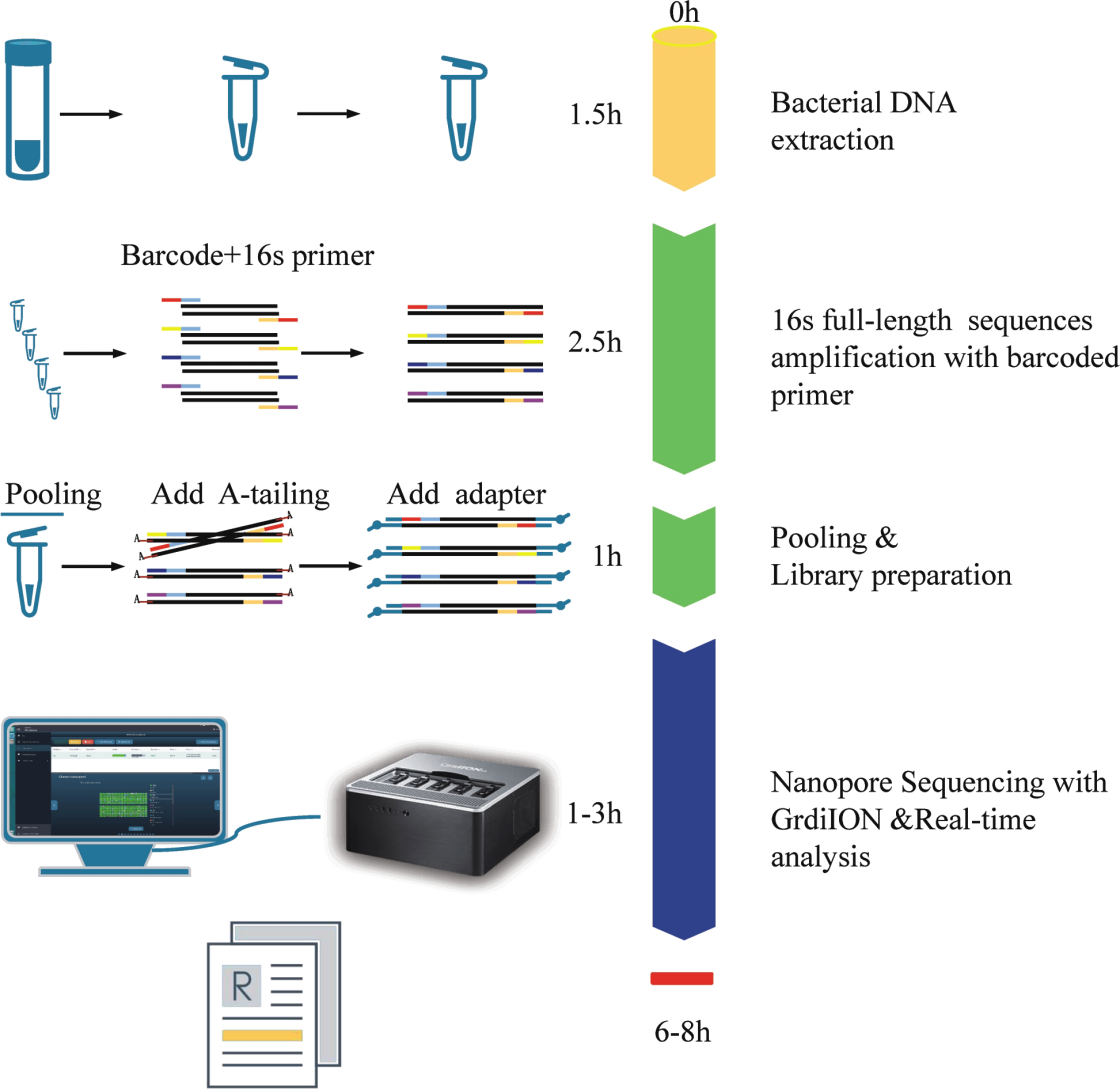
The process and schematic representation of NB16S-seq. The turnaround time was approximately 6~8 h from sample collection to results.

The NB16S-seq workflow is shown in **Figure 1**. The report time was 8 hours after receiving the BALF sample and could be shortened to 6 hours. This was significantly shorter than the time of culture and NGS. Rapid diagnosis may enhance the early identification of pathogens and contribute to the successful treatment of severe infection.

### Evaluating the NB16S-seq test with reference strains, clinical isolates and BALF specimens

The NB16S-seq results for these 21 bacterial strains are shown in **Table 1**. All bacterial species in **Table 1** were correctly detected with an abundance of ≥ 99%, suggesting that the NB16S-seq is accurate in the detection of common pathogens of pulmonary infection.

**Table 1:** Evaluating the NB16S-seq test with 21 common respiratory pathogens. Seven reference strains and 14 clinical isolates were used to verify the detection results of the NB16S-seq.

Species	Strain	Code	NB16S-seq results		
			Species	Reads (n)	Abundance (%)
S. pneumoniae	ATCC49619		S. pneumoniae	31628	99.82
H. influenzae	ATCC49247		H. influenzae	37192	99.96
M. catarrhalis	211227241		M. catarrhalis	35127	99.99
S. aureus	ATCC25923		S. aureus	30350	99.87
S. pyogenes	ATCC19615		S. pyogenes	35378	99.97
E. coli	ATCC25922		E. coli	33651	99.6
K. pneumoniae	211227130		K. pneumoniae	32330	99.5
K. aerogenes	21WJ8254	210919133	K.a aerogenes	32505	99.97
S. marcescens	21WJ8334	211223048	S. marcescens	36867	99.85
S. enterica	ATCC14028		S. enterica	28901	99.71
N. meningitidis	21WJ8288	211029149	N. meningitidis	42568	99
L.monocytogenes	21WJ8301	2111111189	L.monocytogenes	30327	99.98
P. aeruginosa	ATCC27853		P. aeruginosa	10402	99.88
S. maltophilia	21WJ8338	211223215	S. maltophilia	11331	99.81
A. baumannii	211226029		A. baumannii	13591	99.6
A. johnsonii	21WJ8155	210615244	A. johnsonii	8891	99.44
E. faecalis	21WJ8320	211206091	E. faecalis	46805	99.91
E. faecium	211227058		E. faecium	37730	99.91
S. mitis	21WJ8314	211124076	S. mitis	27988	99.43
M. osloensis	21WJ8171	210706090	M. osloensis	35673	99.96
B. cepacia	21WJ8247	210906065	B. cepacia	11921	99.83

The accuracy rate for bacterial species with an abundance ≥ 99% was 100%.

**Figure 2:**
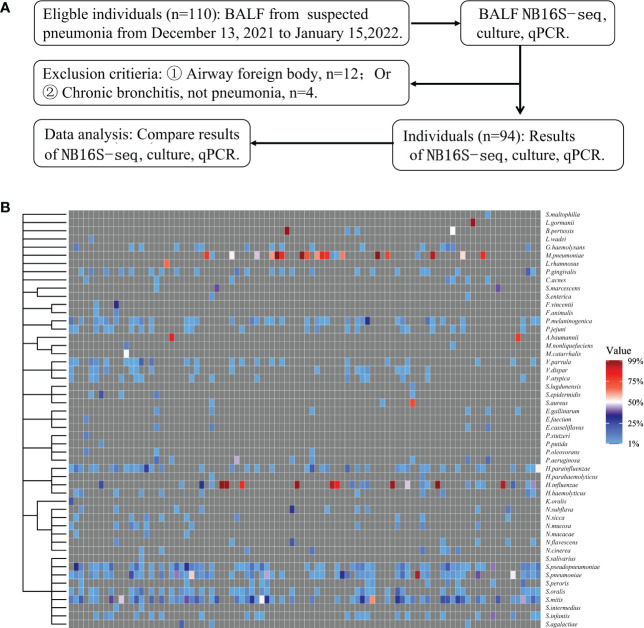
Evaluation of NB16S-seq in the etiological diagnosis of pediatric pneumonia **(A)**: BALF form pediatric pneumonia tested with NB16S-seq, culture or qPCR in our hospital between November 2021 and January 2022. **(B)**. Heatmap of bacteria abundance ≥1.0% in 94 BALF samples tested by NB16S-seq. Each column in the **(C)** indicates a sample. Formula of abundance = (number of sequences for a species/total number of sequences in the specimen) ×100%. The value indicates abundance, and different colors of the value bar represent the different NB16S-seq abundances.

Among the 110 eligible BALF samples, 94 met the criteria (**Figure 2A**). The demographic and clinical characteristics of the patients are shown in **Table 2**, including 88 cases of community acquired pneumonia, 6 cases of hospital acquired pneumonia (HAP), 15 cases of acute respiratory distress syndrome, 20 cases with ICU admission, 1 case of progression, 2 cases of death, and 91 cases of improvement or recovery. The NB16S-seq results regarding bacteria and their abundances are shown in a heatmap (**Figure 2B**, detail data seen in **Supplementary Table 2**). The heatmap shows many commensal organisms, fewer opportunistic pathogens, and various abundant common pathogens. This may be related to the following: ① opportunistic pathogens are often isolated in HAP, and only 6 HAP children (6.4%) were enrolled; ② commensal organisms exist in the oropharynx, which might contaminate BALF specimens; and ③ eighty-eight patients (93.6%) received antibiotics before sampling, potentially mitigating pathogen abundance.

**Table 2:** Demographic and clinical characteristics of the 94 pediatric pneumonia patients.

Variable	Pediatric pneumonia, n=94
Age, Median (Interquartile,IQR), month	49.02 (16.58-88.90)
Male, n (%)	45 (47.9%)
Diagnosis at admission, n (%)	
CAP	88 (93.6%)
HAP	6 (6.4%)
ARDS	15 (16.0%)
Symptoms	
Cough	94 (100%)
Fever	51 (54.3%)
Dyspnea	14 (14.9%)
Received antibiotic before sequencing, n (%)	88 (93.6%)
Underlying disease, n (%)	22 (23.4%)
Laboratory findings	
White blood cell, Median (IQR), ×10^9^/L	10.17 (7.71-14.54)
Neutrophils, Median (IQR), ×10^9^/L	5.78 (3.63-8.58)
CRP, Median (IQR), mg/L	11.47 (0.5775-50.7)
PCT, Median (IQR), ng/ml	0.123 (0.065-0.49)
Pneumonia confirmed by image, n (%)	94 (100%)
Chest X-ray, pneumonia, n (%)	64 (68.1%)
Pulmonary CT, n (%)	92 (97.9%)
Consolidation or atelectasis	14 (14.9%)
Lung abscess or cavity	2 (2.1%)
Pleural inflammation	11 (11.7%)
Airway malformation	13 (13.8%)
Duration from admission to sequencing, Median (IQR), days	3 (1-5)
Invasive mechanical ventilation, n (%)	7 (7.4%)
Length of hospital stay, Median (IQR), days	8 (6-11)
ICU admission, n (%)	19 (20.2%)
Length of ICU stay, Median (IQR),days	20 (8-44)
Outcome, n (%)	
Improvement or recovery	91 (96.8%)
Progress	1 (1.1%)
Death	2 (2.1%)

### Exploring the appropriate NB16S-seq threshold to identify pathogen

**Figure 3:**
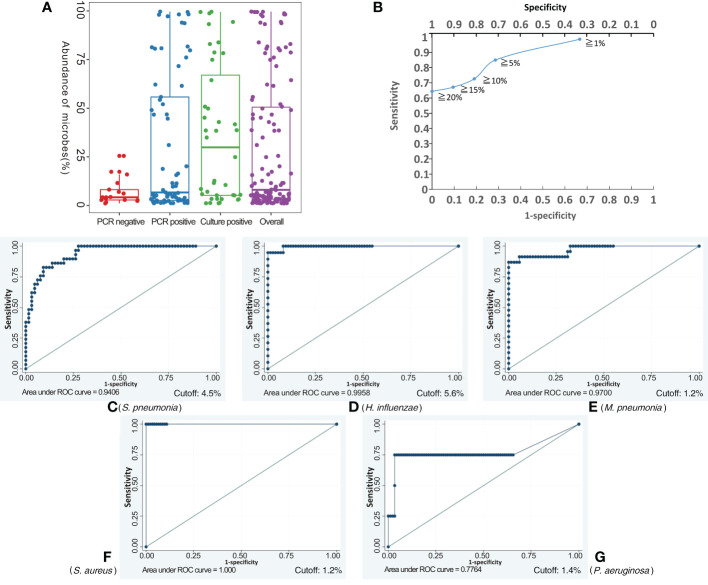
Exploring the appropriate NB16S-seq threshold to identify pathogenic bacteria. The relationship between BALF NB16S-seq abundance and positive culture, positive qPCR and negative qPCR results **(A)**. Correlation between NB16S-seq abundance threshold and sensitivity and specificity of BALF pathogen detection **(B)**. For common respiratory pathogens, an NB16S-seq abundance ≥ 5% is an appropriate criterion to designate a sample positive for a pathogen **(C-G)**. The relationship between the NB16S-seq abundance threshold and the sensitivity and specificity for different strains, *S. pneumonia* **(C)**, *H. influenza* **(D)**, *M. pneumonia* **(E)**, *S. aureus* **(F)**, and *P. aeruginosa* **(G)**. A value of 4.5% was an appropriate cutoff for *S. pneumonia* **(C)**, 5.6% for *H. influenzae* **(D)**, 1.2% for *M. pneumonia* **(E)** and *S. aureus* **(F)**, and 1.4% for *P. aeruginosa* **(G)**.

Applying the BALF culture or qPCR results as the gold standard, BALF NB16S-seq results were analyzed (details in **Supplementary Table 3**). Seventy-one (75.5%) of the 94 BALF specimens were positive according to the gold standard, 25 (26.6%) were culture positive, and 52 (55.3%) were qPCR positive. The number of samples containing 0, 1, 2, 3 and 5 pathogenic bacteria with detected abundance ≥ 1% for each bacterium were 7, 54, 28, 4 and 1, respectively. The relationship between NB16S-seq abundance and positive culture, positive PCR and negative PCR is shown in **Figure 3A**, and the median abundances were 29.9%, 6.7% and 4.2%, respectively. The abundance on bacterial NB16S-seq was closely related to the positive culture and qPCR results. The NB16S-seq abundance of three-quarters of the BALF with positive culture and positive qPCR was ≥5.2% and 2.7%, respectively.

With the increase in the specimens’ NB16S-seq abundance threshold to 1%, 5%, 10%, 15% and 20%, the test sensitivity decreased gradually to 98.6%, 84.9%, 72.6%, 67.1% and 64.4%, respectively, and the test specificity increased progressively to 33.3%, 71.4%, 81.0%, 90.5% and 100.00%, respectively (see **Figure 3B**). If a low NB16S-seq abundance of common pathogens was detected in the BALF, the relationship between the bacteria and pulmonary infection was further evaluated, as these might have been colonizing bacteria. Therefore, for all common respiratory pathogens with an NB16S-seq abundance ≥ 1%, a cut-off value ≥ 5% was applied because at this cut-off the Youden Index (84.9% + 71.4% - 100% = 56.3%) was the largest.

According to the gold standard, *S. pneumonia* was detected 29 times, *M. pneumoniae* 23 times, *H. influenzae* 18 times, *P. aeruginosa* 4 times, *A. baumannii* thrice, both *S. aureus* and *B. pertussis* twice, along with *M. catarrhalis*, *S. agalactiae*, *L. gormanii*, and *S. marcescens* being detected once in 94 BALF specimens (details in **Supplementary Table 3**). When the cut-off value of specimens’ NB16S-seq abundance was the same, the sensitivity and specificity of each pathogen were different (**Figure 3**). The relationship between the NB16S-seq abundance threshold, together with the sensitivity and specificity of common strains was analyzed (**Table 3**). Accordingly, 4.5% was an appropriate cut-off for *S. pneumonia* (**Figure 3C**), 5.6% for *H. influenzae* (**Figure 3D**), 1.2% for *M. pneumonia* (**Figure 3E**) and *S. aureus* (**Figure 3F**), and 1.4% for *P. aeruginosa* (**Figure 3G**). The appropriate cutoff value of *S. pneumonia* and *H. influenzae* was higher than that of *M. pneumonia, S. aureus* and *P.aeruginosa*, which may be related to the oropharyngeal colonization of *H. influenzae* and *S. pneumonia*, while the possibility of colonization of the other three bacteria was low.

**Table 3:** The relationship between the NB16S-seq abundance threshold and the sensitivity and specificity for different strains according to the gold standard test (either culture or PCR) results.

	Cutoff (%)	Sensitivity (%)	Specificity (%)	AUC
*S. pneumonia*	1.3	100	60.0	0.9406
	4.5	82.7	90.8	
	10.0	44.8	98.5	
*H. influenzae*	1.2	94.7	92.0	0.9958
	5.6	94.7	98.7	
	10.5	84.2	100	
*M. pneumonia*	1.2	87.0	98.6	0.9700
	5.2	82.6	100.0	
*S. aureus*	1.2	100.0	98.9	1.000
	2.1	100.0	100.0	
	74.9	50.0	100.0	
*P. aeruginosa*	1.4	75.0	94.4	0.7764
	5.2	50.0	96.7	
	11.4	25.0	96.7	

For common respiratory tract colonizers (such as S. pneumonia and H. influenzae), an NB16S-seq abundance ≥ 4.5% is an appropriate criterion to designate a sample positive for a pathogen; while for bacteria (such as M. pneumonia, S. aureus, and P. aeruginosa) that rarely colonize the respiratory tract, ≥1.2% is an appropriate criterion. AUC, area under the ROC curve.

To optimize the interpretations of NB16S-seq results, an abundance cut-off of 1.0% might be appropriate for organisms that are not typical colonizers of upper respiratory tract (e.g., *M. pneumonia, S. aureus*, and *P. aeruginosa*), and 5.0% for the other eight bacteria that typically colonize the upper respiratory. NB16S-seq abundances were evaluated with these two different cut-off values, the true or false positive, and true or false negative results are shown in **Table 4**, the sensitivity, specificity, positive and negative predictive values were 85.9%, 73.6%, 83.9% and 76.5%, respectively, and after applying two different abundance cut-off values for two different group of bacteria, Youden Index (85.9%+73.6% -100%=59.5%) was higher than that (56.3%) of single 5.0% cutoff value.

**Table 4:** Evaluating BALF NB16S-seq with the gold standard test (either culture or qPCR).

NB16S-seq Result	Gold Standard	No. of Samples	Definition	Category Description	Sensitivity	Specificity	PPV	NPV
Neg (51)	Neg (39)	37	TN	Culture Neg and qPCR ND	85.9%	73.6%	83.9%	76.5%
	Pos (12)	10	FN	Culture Neg, qPCR Pos				
		2	FN	Culture Pos				
Pos (87)	Neg (14)	11	FP	Culture Neg, qPCR Pos				
		2	FP	Culture Pos				
	Pos (73)	44	TP	Culture Neg, PCR Pos				
		24	TP	Culture Pos				

The strains with abundance ≥1% were compared with gold standard. The number of samples containing 0, 1, 2, 3 and 5 pathogenic bacteria with an abundance ≥ 1% was 7, 54, 28, 4 and 1, respectively. Pathogens with negative culture and positive NB16S-seq (abundance ≥5%) results were verified by qPCR. If only one pathogen was detected and its abundance was between 1% and 5%, qPCR verification was also performed. Formula of abundance = (number of sequences for a species/total number of sequences in the specimen) ×100%. To evaluate the NB16S-seq results, the abundance cut-off applied for different bacteria was either 5.0% or 1.0% (1.0% for M. pneumonia, S. aureus, and P. aeruginosa, 5% for the other 8 bacteria). Neg, negative; Pos, positive; TP, true positive; FP, false positive; FP, false positive; FN, false negative; PPV, positive predictive value; NPV, negative predictive value.Eleven species were considered as common respiratory pathogens in this study, *S. pneumoniae*, *H. influenzae*, *M. catarrhalis*, *M. pneumoniae*, *S. aureus*, *S. agalactiae*, *L. gormanii*, *B. pertussis*, *P. aeruginosa*, *S. marcescens*, and *A. baumannii*.

## Discussion

Pneumonia is one of the main causes of death in children under 5 years old (Liu et al., 2015). Timely and appropriate antibiotic treatment could significantly reduce mortality. However, diversity and antibiotic resistance complicates the pathogenic treatment (Yiang et al., 2021). Rapid and sensitive pathogen identification is important. Routine pathogen detection cannot meet clinical needs due to prolonged time period required or limited detection of single or few kinds of pathogens (Holter et al., 2015; Hassibi et al., 2018; Klein et al., 2021).

In recent years, the application of nanopore sequencing technology in pathogen detection has gained much attention. Compared with the NGS, nanopore sequencing technology has the following advantages (Goldberg et al., 2015; Sedlazeck et al., 2018): ① shorter time from sampling to results, ② simpler sequencing library construction, ③ long sequence reading without assembly, and ④ real-time sequencing and analysis. Compared with the previous 16S rDNA nanopore sequencing method, the NB16S-seq has the following advantages: ① reduced the reagent cost for each sample, and ② optimized the experimental procedure.

The NB16S-seq method includes bacterial DNA extraction, 16S rDNA amplification, pooling and library preparation, nanopore sequencing and real-time analysis. The NB16S-seq was verified for 7 reference strains and 14 clinical isolates. The accuracy rate for species was 100%, and their abundance was ≥ 99%. In the process of 16S rDNA amplification and species database comparison, there was no error, and no other species appeared incorrectly. The 16S rDNA is a commonly used molecular clock in bacterial systematic classification. Bacterial universal primers can amplify the 16S rDNA fragments of all bacteria, and the difference in variable regions can be used to distinguish different bacteria. As an increasing number of bacterial 16S rDNA sequences are being determined and included in the NCBI and GTDB databases, it is faster and more convenient to identify species based on the 16S rDNA (Robeson et al., 2021). The similarity rate of 16S rDNA among some species (such as: *S. pneumoniae* and *S. mitis*) is greater than 99%, which intricates the difficulty of species identification (Church et al., 2020; Yatera et al., 2021). Interestingly, the NB16S-seq can effectively distinguish them.

Among the bacteria detected by the NB16S-seq, oropharyngeal-colonizing bacteria were the most common. In particular, the BALF culture reported them as colonizing bacteria, and the NB16S-seq listed them in the appendix results as detected bacteria. These bacteria can cause infective endocarditis, meningitis, and local tissue abscesses. The NGS showed that colonizing anaerobic bacteria in the oropharynx are closely related to lung abscesses (Mu et al., 2021).

Some pathogenic bacteria, such as *S. pneumoniae* and *H. influenzae* may colonize the oropharynx. Furthermore, differentiating commensal from pathogens is complex. However, our results showed the NB16S-seq abundance to be beneficial in distinguishing pathogens from commensals. When the NB16S-seq abundance was increased from 1% to 5%, the specificity also increased from 33.3% to 71.5%, and significantly reduced the false-positive rate. When the abundance extended to 20%, the specificity was 100%. Some pathogenic bacteria, such as *M. pneumoniae*, *S. aureus* and *P. aeruginosa* rarely colonize the oropharynx. Interestingly, they had a high specificity when the abundance was ≥ 1%, implying that the bacteria exist in the BALF.

## Conclusions

We report a rapid pathogen diagnostic method, the NB16S-seq, for use in bacterial pneumonia with an optimized experimental procedure and lower cost. Pathogens can be identified in 6~8 h. For oropharyngeal-colonizing pathogens, an NB16S-seq abundance of 5% is the best cutoff point, and 1% is the best cutoff for pathogens that rarely colonize.

## Data availability statement

The datasets presented in this study can be found in online repositories. The names of the repository/repositories and accession number(s) can be found below: We have uploaded the sequencing to the GSA, Project Number is PRJCA011155, which can be visited in https://ngdc.cncb.ac.cn/bioproject/browse/PRJCA011155.

## Ethics statement

The studies involving human participants were reviewed and approved by the ethics committee of The Children’s Hospital of Zhejiang University of Medicine. Written informed consent to participate in this study was provided by the participants’ legal guardian/next of kin. Written informed consent was obtained from the minor(s)’ legal guardian/next of kin for the publication of any potentially identifiable images or data included in this article.

## Author contributions

YC and LM contributed equally to this work and share first authorship. YC, DL, and WX collected and analyzed medical data of the patients, wrote and revised the manuscript. YZ, ZL, and SZ participated in the treatment of the patients and data collection. LM, SW, DY, YX, YT, XM, MW, and JS participated in the Improved targeting 16S rDNA nanopore sequencing and data analysis. QC and QS contributed to the treatment plan and made a critical revision of the manuscript. All authors contributed to the article and approved the submitted version.
