# Supplementary material for: Improved targeting of the 16S rDNA nanopore sequencing method enables rapid pathogen identification in bacterial pneumonia in children
Source: Front Cell Infect Microbiol. 2023 Jan 9;12:1001607. doi: 10.3389/fcimb.2022.1001607 (PMC9868273; doi:10.3389/fcimb.2022.1001607)
Supplement: Supplementary file 1 [file Table_1.docx]

| Order | Species | Number of strains | Proportion (%) |
| --- | --- | --- | --- |
| The BALF culture results from the Children's Hospital of Zhejiang University School of Medicine, from January 1, 2019 to September 30, 2021, . | | | |
| 1 | S. pneumoniae | 134 | 30.80 |
| 2 | S. aureus | 46 | 10.57 |
| 3 | H. influenzae | 46 | 10.57 |
| 4 | P. aeruginosa | 36 | 8.28 |
| 5 | M.catarrhalis | 33 | 7.59 |
| 6 | A. baumannii complex | 24 | 5.52 |
| 7 | S. maltophilia | 23 | 5.29 |
| 8 | K. pneumoniae | 24 | 5.52 |
| 9 | 1. coli | 16 | 3.68 |
| 10 | B. cepacia | 15 | 3.45 |
| 11 | S. marcescens | 9 | 2.07 |
| 12 | E. cloacae complex | 11 | 2.53 |
| 13 | E. aerogenes | 6 | 1.38 |
| 14 | S. pyogenes | 3 | 0.69 |
| 15 | K. oxytoca | 2 | 0.46 |
| 16 | E. meningealis | 1 | 0.23 |
| 17 | S. lactis | 1 | 0.23 |
| 18 | A. watson | 1 | 0.23 |
| 19 | A. yoelii | 1 | 0.23 |
| 20 | C. gleum | 1 | 0.23 |
| 21 | S. intermedius | 1 | 0.23 |
| 22 | C. fraudi | 1 | 0.23 |
| Respiratory pathogens that cannot be detected by routine culture | | | |
| 23 | M. pneumoniae |  |  |
| 24 | 1. tuberculosis |  |  |

***Supplementary Material***

**Supplementary table 1**: Common pathogens detected in BALF by culture. From January 1, 2019 to September 30, 2021, the BALF culture results from the Children's Hospital of Zhejiang University School of Medicine, the duplicate strains of the same patient were removed.
