# Supplementary material for: Improved targeting of the 16S rDNA nanopore sequencing method enables rapid pathogen identification in bacterial pneumonia in children
Source: Front Cell Infect Microbiol. 2023 Jan 9;12:1001607. doi: 10.3389/fcimb.2022.1001607 (PMC9868273; doi:10.3389/fcimb.2022.1001607)
Supplement: Supplementary file 3 [file Table_3.docx]

Supplementary Table 3.

|  | **BALF NB16S-seq results, abundances (%)** | | | | | | | | | | | **Gold standard** | | | **Evaluation** |
| --- | --- | --- | --- | --- | --- | --- | --- | --- | --- | --- | --- | --- | --- | --- | --- |
| **Pathogen** | **Lg** | **Bp** | **Mp** | **Sm** | **Ab** | **Mc** | **Sau** | **Pa** | **Hi** | **Sp** | **Sag** | **Culture** | | **qPCR** |  |
| **1** | 0.0 | 0.0 | 0.0 | 0.0 | 0.0 | 0.0 | 0.0 | 0.0 | 0.2 | 1.6^&^ | 0.0 | N | ND | | TN |
| **2** | 0.0 | 0.0 | 0.0 | 0.0 | 0.0 | 0.0 | 0.0 | 0.0 | 0.0 | 7.0^#^ | 0.0 | N | Sp (-) | | FP |
| **3** | 0.0 | 0.0 | 0.0 | 0.0 | 0.0 | 0.0 | 0.0 | 0.0 | 0.0 | 4.2^&^ | 0.0 | N | ND | | TN |
| **4** | 0.0 | 0.0 | 0.0 | 0.0 | 0.0 | 0.0 | 0.0 | 0.0 | 0.0 | 0.0 | 0.0 | N | ND | | TN |
| **5** | 0.0 | 0.0 | 0.1 | 0.0 | 0.0 | 0.0 | 0.0 | 0.8 | 1.0^&^ | 8.2^#^ | 0.0 | N | Sp (-) | | TN,FP |
| **6** | 0.0 | 0.0 | 0.0 | 0.0 | 0.0 | 0.0 | 0.0 | 0.0 | 0.5 | 2.9^&^ | 0.0 | N | ND | | TN |
| **7** | 0.0 | 0.1 | 0.0 | 0.0 | 0.0 | 0.0 | 0.0 | 0.0 | 25.5^#^ | 0.0 | 0.0 | N | Hi (-) | | FP |
| **8** | 0.0 | 0.0 | 0.0 | 0.0 | 0.0 | 0.0 | 0.0 | 0.0 | 0.1 | 4.2^&^ | 0.0 | N | ND | | TN |
| **9** | 0.0 | 0.0 | 0.1 | 0.4 | 0.0 | 0.0 | 0.0 | 0.0 | 0.1 | 6.2^#^ | 0.0 | N | Sp (-) | | FP |
| **10** | 0.0 | 0.0 | 0.0 | 0.0 | 0.0 | 0.0 | 0.0 | 0.1 | 0.0 | 0.5 | 0.0 | N | ND | | TN |
| **11** | 0.0 | 0.0 | 0.0 | 0.0 | 0.0 | 0.1 | 0.0 | 0.9 | 0.0 | 0.4 | 0.0 | N | ND | | TN |
| **12** | 0.0 | 0.0 | 0.0 | 0.0 | 0.0 | 49.8* | 0.0 | 0.0 | 0.0 | 0.8 | 0.0 | Mc | ND | | TP |
| **13** | 0.0 | 0.0 | 0.0 | 0.0 | 0.0 | 0.4 | 0.0 | 0.1 | 0.0 | 24.9* | 0.0 | Sp | ND | | TP |
| **14** | 0.0 | 0.0 | 0.0 | 0.0 | 0.0 | 0.0 | 0.0 | 0.0 | 0.0 | 4.4^&^ | 0.0 | N | ND | | TN |
| **15** | 0.0 | 0.0 | 0.0 | 0.0 | 0.0 | 0.0 | 0.0 | 0.0 | 0.0 | 2.7^&^ | 0.0 | N | ND | | TN |
| **16** | 0.0 | 0.0 | 0.0 | 0.0 | 0.0 | 0.0 | 0.0 | 0.0 | 0.4 | 0.0 | 0.0 | N | ND | | TN |
| **17** | 0.0 | 0.7 | 0.1 | 0.0 | 0.0 | 0.0 | 0.0 | 0.0 | 0.0 | 2.8^&^ | 0.0 | N | ND | | TN |
| **18** | 0.0 | 0.0 | 0.5 | 1.1^#^ | 0.0 | 0.0 | 0.0 | 15.9^#^ | 0.5 | 0.0 | 0.0 | N | Pa (-) | | FP |
| **19** | 0.0 | 0.0 | 0.1 | 0.0 | 0.0 | 0.0 | 0.0 | 0.0 | 0.0 | 11.6^#^ | 0.0 | N | Sp (-) | | FP |
| **20** | 0.0 | 0.0 | 0.0 | 0.0 | 0.0 | 0.0 | 0.0 | 0.0 | 0.0 | 0.0 | 0.0 | N | ND | | TN |
| **21** | 0.1 | 0.0 | 0.0 | 0.0 | 83.8* | 0.0 | 0.0 | 3.4* | 0.1 | 3.4^&^ | 0.0 | Ab, Pa | ND | | TP |
| **22** | 0.0 | 0.0 | 0.0 | 0.0 | 0.0 | 0.0 | 0.0 | 0.0 | 0.0 | 41.7* | 0.0 | Sp | ND | | TP |
| **23** | 0.0 | 0.0 | 0.0 | 0.0 | 0.0 | 0.0 | 0.0 | 0.0 | 3.2^&^ | 6.5* | 20.2* | N | Sp(+),Sag(+) | | TN,TP |
| **24** | 0.0 | 0.0 | 0.0 | 0.0 | 0.0 | 0.0 | 0.0 | 0.0 | 0.0 | 2.6ǂ | 0.0 | N | Sp (+) | | FN |
| **25** | 0.0 | 0.3 | 0.0 | 0.0 | 0.0 | 0.0 | 0.0 | 0.0 | 0.0 | 55.8* | 0.0 | N | Sp (+) | | TP |
| **26** | 0.0 | 0.1 | 0.0 | 0.0 | 0.0 | 0.0 | 0.0 | 0.0 | 0.0 | 9.7* | 0.1 | N | Sp (+) | | TP |
| **27** | 0.0 | 0.0 | 0.0 | 0.0 | 0.0 | 0.0 | 0.0 | 0.0 | 31.1* | 5.5* | 0.0 | N | Sp(+), Hi (+) | | TP |
| **28** | 0.0 | 0.0 | 78.7* | 0.0 | 0.0 | 0.0 | 0.0 | 0.0 | 0.0 | 11.6* | 0.0 | Sp | Mp (+) | | TP |
| **29** | 0.0 | 0.0 | 1.2* | 0.0 | 0.0 | 0.0 | 1.2^#^ | 0.7 | 3.3^&^ | 4.9^&^ | 4.5^&^ | N | Mp (+) | | TP, FP,TN |
| **30** | 0.0 | 0.0 | 0.0 | 38.4* | 0.0 | 0.0 | 0.0 | 0.0 | 0.0 | 0.7 | 0.0 | Sm | ND | | TP |
| **31** | 0.0 | 0.0 | 0.0 | 0.0 | 0.0 | 0.0 | 0.0 | 0.0 | 99.5* | 0.0 | 0.0 | Hi | ND | | TP |
| **32** | 0.0 | 0.0 | 0.0 | 0.0 | 0.0 | 0.0 | 0.0 | 0.0 | 98.2* | 0.1 | 0.0 | N | Hi (+) | | TP |
| **33** | 0.0 | 0.0 | 52.0* | 0.0 | 0.0 | 0.0 | 0.0 | 0.0 | 0.0 | 1.3^&^ | 0.0 | N | Mp (+) | | TP,TN |
| **34** | 0.0 | 0.0 | 0.0 | 0.0 | 0.0 | 0.0 | 0.0 | 45.1* | 0.0 | 0.7 | 0.0 | Pa | ND | | TP |
| **35** | 0.0 | 0.0 | 0.0 | 0.0 | 0.0 | 0.0 | 0.0 | 0.0 | 80.8* | 0.6 | 0.0 | N | Hi (+) | | TP |
| **36** | 0.0 | 0.0 | 0.0 | 0.0 | 0.0 | 0.0 | 0.0 | 0.0 | 0.0 | 6.0* | 0.0 | N | Sp (+) | | TP |
| **37** | 0.0 | 0.0 | 0.0 | 0.0 | 0.0 | 0.0 | 0.0 | 0.1 | 0.1 | 11.5* | 0.0 | N | Sp (+) | | TP |
| **38** | 0.0 | 0.0 | 46.7* | 0.0 | 0.0 | 0.0 | 0.0 | 0.0 | 0.0 | 0.4 | 0.0 | N | Mp (+) | | TP |
| **39** | 0.0 | 0.0 | 0.0 | 0.0 | 0.0 | 0.0 | 0.0 | 0.0 | 0.0 | 2.9ǂ | 0.0 | N | Sp (+) | | FN |
| **40** | 0.0 | 0.0 | 0.1 | 0.0 | 0.0 | 0.0 | 0.0 | 0.0 | 0.0 | 44.6* | 0.0 | N | Sp (+) | | TP |
| **41** | 0.0 | 0.0 | 61.5* | 0.0 | 0.0 | 0.0 | 0.0 | 0.0 | 0.0 | 1.3^&^ | 0.0 | N | Mp (+) | | TP,TN |
| **42** | 0.0 | 0.0 | 99.6* | 0.0 | 0.0 | 0.0 | 0.0 | 0.0 | 0.0 | 0.0 | 0.0 | N | Mp (+) | | TP |
| **43** | 0.0 | 0.0 | 81.3* | 0.0 | 0.0 | 0.0 | 0.0 | 0.0 | 0.0 | 1.3^&^ | 0.0 | N | Mp (+) | | TP,TN |
| **44** | 0.0 | 98.8* | 0.0 | 0.0 | 0.0 | 0.0 | 0.0 | 0.0 | 0.0 | 0.2 | 0.0 | Bp | ND | | TP |
| **45** | 0.0 | 0.0 | 0.0 | 0.0 | 0.0 | 0.0 | 0.0 | 3.2* | 0.0 | 0.2 | 0.0 | Pa | ND | | TP |
| **46** | 0.0 | 0.0 | 0.1 | 0.0 | 0.0 | 0.0 | 0.0 | 0.1 | 98.1* | 0.0 | 0.0 | N | Hi (+) | | TP |
| **47** | 0.0 | 0.0 | 98.2* | 0.0 | 0.0 | 0.0 | 0.0 | 0.1 | 0.0 | 0.0 | 0.0 | N | Mp (+) | | TP |
| **48** | 0.0 | 0.0 | 71.7* | 0.0 | 0.0 | 0.0 | 0.0 | 0.1 | 0.0 | 0.6 | 0.0 | N | Mp (+) | | TP |
| **49** | 0.0 | 0.0 | 0.2 | 0.0 | 0.0 | 0.1 | 0.0 | 5.2* | 0.1 | 2.7^&^ | 0.0 | N | Pa (+) | | TP,TN |
| **50** | 0.0 | 0.0 | 62.2* | 0.0 | 0.0 | 0.0 | 0.0 | 0.1 | 0.0 | 2.4^&^ | 0.0 | N | Mp (+) | | TP,TN |
| **51** | 0.0 | 0.0 | 81.7* | 0.0 | 0.0 | 0.0 | 0.0 | 0.0 | 0.0 | 1.5^&^ | 0.0 | N | Mp (+) | | TP,TN |
| **52** | 0.0 | 0.0 | 79.7* | 0.0 | 0.0 | 0.0 | 0.0 | 0.0 | 0.0 | 0.5 | 0.0 | N | Mp (+) | | TP |
| **53** | 0.0 | 0.0 | 5.2* | 0.0 | 0.0 | 0.0 | 0.0 | 0.2 | 93.2* | 0.0 | 0.0 | Hi | Mp (+) | | TP |
| **54** | 0.0 | 0.0 | 1.2^#^ | 0.0 | 0.0 | 0.0 | 0.0 | 0.4 | 83.0* | 1.3^&^ | 0.0 | Hi | ND | | FP, TP,TN |
| **55** | 0.0 | 0.0 | 64.4* | 0.0 | 0.0 | 0.0 | 0.0 | 0.0 | 0.0 | 34.9* | 0.0 | Sp | Mp (+) | | TP |
| **56** | 0.0 | 4.2^&^ | 0.0 | 0.0 | 0.0 | 0.0 | 0.0 | 0.1 | 0.0 | 15.6* | 0.0 | N | Sp (+) | | TN,TP |
| **57** | 0.0 | 0.0 | 0.1 | 0.0 | 0.0 | 0.0 | 0.0 | 0.0 | 12.7* | 0.8 | 0.0 | Hi | ND | | TP |
| **58** | 0.0 | 2.0^&^ | 0.2 | 0.0 | 0.0 | 0.0 | 0.0 | 0.1 | 0.1 | 5.9* | 0.0 | N | Sp (+) | | TN,TP |
| **59** | 0.0 | 0.0 | 0.5 | 0.1 | 0.0 | 0.0 | 0.0 | 0.1 | 8.5* | 5.8^#^ | 0.1 | Hi | Sp (-) | | TP, FP |
| **60** | 0.0 | 0.0 | 0.2 | 0.0 | 0.0 | 0.0 | 0.0 | 0.0 | 10.5* | 5.2* | 0.0 | Hi, Sp | ND | | TP |
| **61** | 0.0 | 0.0 | 0.1 | 0.0 | 0.0 | 0.0 | 0.0 | 0.0 | 0.2 | 2.6ǂ | 0.0 | N | Sp (+) | | FN |
| **62** | 0.0 | 0.0 | 99.4* | 0.0 | 0.0 | 0.0 | 0.0 | 0.0 | 0.0 | 0.0 | 0.0 | N | Mp (+) | | TP |
| **63** | 0.0 | 0.0 | 0.4 | 0.0 | 0.0 | 0.0 | 2.1* | 0.0 | 0.1 | 0.1 | 0.0 | N | Sau (+) | | TP |
| **64** | 0.0 | 0.0 | 76.1* | 0.0 | 0.0 | 0.0 | 0.0 | 0.1 | 0.0 | 0.9 | 0.0 | N | Mp (+) | | TP |
| **65** | 0.0 | 0.0 | 0.0 | 0.0 | 0.0 | 0.0 | 0.0 | 0.1 | 97.2* | 0.1 | 0.0 | N | Hi (+) | | TP |
| **66** | 0.0 | 0.0 | 0.0 | 0.0 | 0.0 | 0.0 | 0.0 | 0.0 | 0.0 | 4.1ǂ | 0.0 | N | Sp (+) | | FN |
| **67** | 0.0 | 0.0 | 0.2ǂ | 0.0 | 0.0 | 0.0 | 0.0 | 0.0 | 0.0 | 16.4* | 0.0 | N | Mp(+),Sp(+) | | FN, TP |
| **68** | 0.0 | 0.0 | 0.0 | 0.0 | 0.0 | 0.0 | 0.0 | 0.0 | 46.8* | 1.2^&^ | 0.0 | N | Hi (+) | | TP |
| **69** | 0.0 | 0.0 | 0.0 | 0.0 | 0.0 | 0.0 | 74.9* | 1.4^#^ | 0.0 | 0.3 | 0.0 | Sau | ND | | TP, FP |
| **70** | 0.0 | 0.0 | 5.4* | 0.0 | 0.0 | 0.0 | 0.0 | 0.0 | 0.0 | 94.3* | 0.0 | Sp | Mp (+) | | TP |
| **71** | 0.0 | 0.0 | 0.0 | 0.0 | 0.0 | 0.0 | 0.0 | 0.3 | 7.1* | 3.3^&^ | 0.0 | Hi | ND | | TP,TN |
| **72** | 0.0 | 0.0 | 0.0 | 0.0 | 0.0 | 0.0 | 0.0 | 0.0 | 1.2^&^ | 6.7* | 0.0 | N | Sp (+) | | TN,TP |
| **73** | 0.0 | 0.0 | 93.7* | 0.0 | 0.0 | 0.0 | 0.0 | 0.0 | 0.0 | 0.3 | 0.0 | N | Mp (+) | | TP |
| **74** | 0.0 | 0.0 | 0.0ǂ | 0.0 | 0.0 | 0.0 | 0.0 | 0.0 | 99.5* | 0.0 | 0.0 | Hi | Mp (+) | | FN, TP |
| **75** | 0.0 | 0.0 | 0.0 | 0.0 | 0.0 | 0.0 | 0.0 | 0.0 | 0.0 | 4.8ǂ | 0.0 | N | Sp (+) | | FN |
| **76** | 0.0 | 0.0 | 0.0 | 0.0 | 0.0 | 0.0 | 0.0 | 0.9 | 0.0 | 4.5ǂ | 0.0 | N | Sp (+) | | FN |
| **77** | 0.0 | 50.8* | 0.0 | 0.0 | 0.0 | 0.0 | 0.0 | 0.1 | 0.2 | 5.5* | 0.1 | Bp | Sp (+) | | TP |
| **78** | 0.0 | 0.0 | 0.1 | 0.0 | 0.0 | 0.1 | 0.1 | 0.4 | 0.0 | 2.3ǂ | 0.2 | N | Sp (+) | | FN |
| **79** | 0.0 | 0.0 | 54.3* | 0.0 | 0.0 | 0.0 | 0.0 | 0.0 | 0.0 | 1.4^&^ | 0.0 | N | Mp (+) | | TP |
| **80** | 0.1 | 0.3 | 0.0 | 0.0 | 0.1 | 0.0 | 0.0 | 11.4^#^ | 5.6^#^ | 0.4 | 2.4^&^ | N | Pa (-),Hi(-) | | FP,TN |
| **81** | 97.9* | 0.0 | 0.0 | 0.0 | 0.0 | 0.0 | 0.0 | 0.0 | 0.0 | 0.0 | 0.0 | N | Lg (+) | | TP |
| **82** | 0.0 | 0.0 | 0.0 | 0.0 | 0.0 | 0.0 | 0.0 | 0.0 | 1.2ǂ | 38.6* | 0.0 | Sp, Hi | ND | | FN, TP |
| **83** | 0.0 | 0.0 | 80.6* | 0.0 | 0.0 | 0.0 | 0.0 | 0.0 | 3.9^&^ | 0.1 | 0.0 | N | Mp (+) | | TP,TN |
| **84** | 0.0 | 0.0 | 0.0 | 0.0 | 0.0 | 0.0 | 0.0 | 17.3^#^ | 0.0 | 0.0 | 0.0 | N | Pa (-) | | FP |
| **85** | 0.0 | 0.0 | 0.0 | 0.3 | 0.0 | 0.0 | 0.0 | 0.0 | 0.0 | 0.1 | 0.0 | N | ND | | TN |
| **86** | 0.0 | 0.0 | 0.0 | 38.7^#^ | 0.0ǂ | 0.0 | 0.0 | 0.0 | 0.0 | 0.7 | 0.0 | Ab | ND | | FP, FN |
| **87** | 0.0 | 0.0 | 0.0 | 0.0 | 0.0 | 0.0 | 0.0 | 0.0 | 94.0* | 0.0 | 0.0 | N | Hi (+) | | TP |
| **88** | 0.0 | 0.0 | 0.0 | 0.0 | 0.0 | 0.0 | 0.0 | 0.1 | 0.0 | 0.0 | 0.0 | N | ND | | TN |
| **89** | 0.0 | 0.0 | 0.0 | 0.0 | 0.0 | 0.0 | 0.0 | 0.6 | 18.8* | 49.1* | 0.0 | N | Sp(+),Hi(+) | | TP |
| **90** | 0.0 | 0.0 | 0.0 | 0.0 | 78.3* | 0.0 | 0.0 | 0.3 | 0.0 | 0.1 | 0.0 | Ab | ND | | TP |
| **91** | 0.0 | 0.0 | 0.2 | 0.0 | 0.0 | 0.0 | 0.0 | 0.0 | 0.1 | 3.6^&^ | 0.0 | N | ND | | TN |
| **92** | 0.0 | 0.0 | 0.0ǂ | 0.0 | 0.0 | 0.0 | 0.0 | 0.0 | 1.9^&^ | 7.9* | 0.0 | N | Mp(+),Sp(+) | | FN, TN,TP |
| **93** | 0.0 | 0.0 | 0.1 | 0.0 | 0.0 | 0.0 | 0.0 | 0.0 | 42.9* | 3.0 | 0.0 | Hi | ND | | TP |
| **94** | 0.0 | 0.0 | 0.0 | 0.0 | 0.0 | 0.0 | 0.0 | 0.0 | 0.0 | 10.0* | 0.0 | N | Sp (+) | | TP |

Supplementary Table 3: Comparing BALF NB16S-seq results (abundances, %) with the gold standard test (culture or qPCR) results. The strains with abundance ≥1% were evaluated with gold standard. The number of samples containing 0, 1, 2, 3 and 5 pathogenic bacteria with detected abundance ≥ 1% for each bacterium was 7, 54, 28, 4 and 1, respectively. Eleven species were defined as common respiratory pathogens in this study, namely, *S. pneumoniae* (Sp), *H. influenzae* (Hi), *M. catarrhalis* (Mc), *M. pneumoniae* (Mp), *S. aureus* (Sau), *S. agalactiae* (Sag), *L. gormanii* (Lg), *B. pertussis* (Bp), *P. aeruginosa* (Pa), *S. marcescens* (Sm), and *A. baumannii* (Ab). Pathogens with negative culture and positive NB16S-seq (abundance ≥5%) results were verified by qPCR. If only one pathogen was detected and its abundance was between 1% and 5%, qPCR verification was also performed. Formula of abundance = (number of sequences for a species / total number of sequences in the specimen) ×100%. To evaluate the NB16S-seq detection results, the appropriate cutoff for different bacteria was 5.0% or 1.0% (1.0% for *M. pneumonia, S. aureus,* and *P. aeruginosa*, 5% for the other 8 bacteria). ND, not done; N, negative; &, true negative (TN); *, true positive (TP); ǂ, false negative (FN); #, false positive (FP).
